# Case of Sympathetic Puerperal Convulsion

**Published:** 1823-08

**Authors:** George Jewel


### Art. IV.—*Case of sympathetic Puerperal Convulsion.* By George Jewel, Esq.

I am induced to detail the following cases of puerperal convulsions, not for the purpose of eliciting any new theory as to their nature or treatment, but solely with a view of directing the attention of the profession to those puerperal diseases frequently dependent on sympathy.

Mrs. R., a robust healthy woman, thirty years of age, had been in tedious labour three days, of her first child. About four hours previous to my arrival, she was seized with a convulsive paroxysm, which returned at intervals of about an hour and a half, with increasing energy. On examination, I found the os uteri dilated; but the pains, as is usual in similar cases, had diminished so as to be scarcely perceptible. There being great fullness in the vessels of the head, I took sixteen ounces of blood, *pleno rivo*, from the arm. Pressure was made on the sides of the uterus, which produced contraction, and the labour appeared in progress; and, after the succeeding paroxysm, I had the satisfaction of finding the head of the child low in the pelvis. Here it rested some time; and, being disappointed in expulsion of the fœtus taking place as I had anticipated, and the next fit resuming its original violence, I introduced the forceps and delivered without loss of time, which was soon and easily accomplished. The child was dead. The patient had no return of the fits after delivery, and her recovery was as favourable as could be expected.

The wife of another person, a strong healthy women, ætat. thirty-three, who had been on terms of intimacy and friendship with the former patient from childhood, and married about the same period, being anxious for the welfare of her friend, often made enquiries at the house respecting her case; but, hearing of her pregnancy, I forbad her coming into the chamber; notwithstanding which, during my absence from it, she entered, and, approaching the bedside of her friend, remained there a few seconds, just at the termination of a severe paroxysm. A month had elapsed when I accidentally saw her, and I was pleased to find the case of her friend had not made the slightest impression on her mind: on the contrary, she was particularly cheerful, and anticipated the result of her own case with confidence. About a week after, I was sent for to attend in her accouchement; but, being several miles distant at the time, I could not reach her before the expiration of three or four hours. In the mean time, a second servant was dispatched, who informed me his mistress was in a fit. On my arrival, I found her in a strong convulsive paroxysm, and, with the exception of the duration of labour, (for in this instance it was far advanced,) the return and continuance of the paroxysm resembled precisely that of the former patient's. The head of the child had been resting on the perineum two hours, and, there being no obstacle to the application of the forceps, she was speedily delivered; after which the convulsions ceased, and her recovery, though tardy, was complete.

Now, although I do not mean positively to assert that the last case might not have been produced by some accidental cause, at the same time it appears to me very evident to have been the consequence of a morbid impression received on the brain during the time of her witnessing the frightful and distressing state of her friend, although not brought into action until the system was again excited by some fresh irritating cause. Previous to parturition taking place, there was no fullness of the vessels, no irritability, nor, in short, any symptom denoting the approach of this most formidable disease.

It has been before observed, in a celebrated compendium of Midwifery, that, when an ewe lambs, the careful shepherd immediately separates her from the flock, fearing the circumstance might produce premature parturition in the others: so is it in the human subject, that this sympathetic feeling, so very acute in pregnant women, is capable of producing convulsions, premature labour, and even death; and that these cases do occur when probably little suspected, there can, I think, be little doubt of. Not only the above case, but several others, of perhaps a less interesting nature, have fallen under my observation in the course of practice; and I cannot but suggest (and it must appear obvious to every practitioner in midwifery,) the propriety of never permitting a pregnant woman to enter into a lying-in chamber.

30, *Gerrard-street, Soho.*

